# Retraction Note: the association between depression, socio-economic factors and dietary intake in mothers having primary school children living in Rey, South of Tehran, Iran

**DOI:** 10.1186/2251-6581-12-21

**Published:** 2013-05-14

**Authors:** Moloud Payab, Ahmad-reza Dorosty Motlagh, Mohammadreza Eshraghian, Reza Rostami, Fereydoun Siassi, Behnood Abbasi, Mehrnaz Ahmadi, Tina Karimi, Mohammad Yoosef Mahjouri, Soroush Seifirad

**Affiliations:** 1 School of Public Health & Institute of Public Health Researches, Tehran University of Medical Sciences, Tehran, Iran; 2 Institute for Psychology and Educational Sciences, University of Tehran, Tehran, Iran; 3 Faculty of Nutrition & Food Technology, Shahid Beheshti University of Medical Science and Health Service, Tehran, Iran; 4 Islamic Azad University, Sciences and Research Branch, Tehran, Iran; 5 Endocrinology and Metabolism Research Center, Tehran University of Medical Sciences, Tehran, Iran

This article [1] has been retracted by the publisher because it was republished in error [2] in the process of journal acquisition. BioMed Central apologize to the authors and readers for the error and any inconvenience caused.
